# Periadventitial β-aminopropionitrile-loaded nanofibers reduce fibrosis and improve arteriovenous fistula remodeling in rats

**DOI:** 10.3389/fcvm.2023.1124106

**Published:** 2023-02-28

**Authors:** Brandon Applewhite, Aavni Gupta, Yuntao Wei, Xiaofeng Yang, Laisel Martinez, Miguel G. Rojas, Fotios Andreopoulos, Roberto I. Vazquez-Padron

**Affiliations:** ^1^Department of Biomedical Engineering, University of Miami, Coral Gables, FL, United States; ^2^Department of Surgery, University of Miami Miller School of Medicine, Miami, FL, United States; ^3^Lewis Katz School of Medicine, Temple University, Philadelphia, PA, United States

**Keywords:** lysyl oxidase, vascular access, perivascular drug delivery, fibrosis, arteriovenous fistula, β-aminopropionitrile, biomaterials

## Abstract

**Background:**

Arteriovenous fistula (AVF) postoperative stenosis is a persistent healthcare problem for hemodialysis patients. We have previously demonstrated that fibrotic remodeling contributes to AVF non-maturation and lysyl oxidase (LOX) is upregulated in failed AVFs compared to matured. Herein, we developed a nanofiber scaffold for the periadventitial delivery of β-aminopropionitrile (BAPN) to determine whether unidirectional periadventitial LOX inhibition is a suitable strategy to promote adaptive AVF remodeling in a rat model of AVF remodeling.

**Methods:**

Bilayer poly (lactic acid) ([PLA)-]- poly (lactic-co-glycolic acid) ([PLGA)] scaffolds were fabricated with using a two-step electrospinning process to confer directionality. BAPN-loaded and vehicle control scaffolds were wrapped around the venous limb of a rat femoral-epigastric AVF during surgery. AVF patency and lumen diameter were followed monitored using Doppler ultrasound surveillance and flow was measured before euthanasia. AVFs were harvested after 21 days for histomorphometry and immunohistochemistry. AVF compliance was measured using pressure myography. RNA from AVF veins was sequenced to analyze changes in gene expression due to LOX inhibition.

**Results:**

Bilayer periadventitial nanofiber scaffolds extended BAPN release compared to the monolayer design (*p* < 0.005) and only released BAPN in one direction. Periadventitial LOX inhibition led to significant increases in AVF dilation and flow after 21 days. Histologically, BAPN trended toward increased lumen and significantly reduced fibrosis compared to control scaffolds (*p* < 0.01). Periadventitial BAPN reduced downregulated markers associated with myofibroblast differentiation including SMA, FSP-1, LOX, and TGF-β while increasing the contractile marker MYH11. RNA sequencing revealed differential expression of matrisome genes.

**Conclusion:**

Periadventitial BAPN treatment reduces fibrosis and promotes AVF compliance. Interestingly, the inhibition of LOX leads to increased accumulation of contractile VSMC while reducing myofibroblast-like cells. Periadventitial LOX inhibition alters the matrisome to improve AVF vascular remodeling.

## Introduction

Since its inception as a routine surgical procedure six decades ago, the establishment of a functional and durable arteriovenous fistula (AVF) has been an elusive task for even the most experienced surgeons. The recurrence of AVF stenosis, before or after access use, poses a tremendous healthcare burden and drastically reduces the quality of life for patients with end stage kidney disease (ESKD). In fact, estimates suggest that only 40% of AVFs mature for dialysis, with a significant portion requiring intervention to facilitate maturation (1). There is still no clinical option to ensure maturation and AVF longevity despite these subpar outcomes.

We previously disclosed a link between postoperative fibrosis and AVF non-maturation (2) which is associated with increased lysyl oxidase (LOX) expression (3). LOX and its analogues, the LOX-like family, catalyze covalent crosslinking of collagen and elastin monomers, the primary constituents of the vascular ECM, to form supramolecular bundles that impart the characteristic biomechanics of healthy vasculature. At the cellular level, ECM properties such as stiffness, composition, and topography influence cell differentiation and migration (4). These signals are either transduced directly *via* integrin-mediated signaling and indirectly *via* sequestration of soluble cytokine mediators. Therefore, LOX may indirectly affect cell behavior by regulating ECM structure. LOX also interacts with extracellular signaling molecules and nuclear transcription factors to directly influence cell phenotype (5). Aberrant LOX activity has been associated with increased in tissue stiffness, fibrosis, and pathological cancer-like microenvironments (3, 6, 7).

Myofibroblasts (MFs) are the premier cellular sources of fibrosis-related ECM components and matrix contraction and are increasingly implicated in surgical restenosis (8–10). Classically, MFs facilitate wound healing by secreting new ECM and forming granulation tissue. However, MFs are dually detrimental to AVF maturation as they contribute to fibrotic remodeling and venous neointimal hyperplasia (VNH), which in combination significantly increase the risk of early failure (2). Vascular MFs arise from diverse lineages *via* differing mechanisms including adventitial fibroblast activation and vascular smooth muscle cell (VSMC) dedifferentiation (11). Differentiation of fibroblasts to MFs is a mechanosensitive process, traditionally associated with increases ECM stiffness (12). Therefore, therapies that attenuate ECM stiffening are a potential strategy to revert the MF phenotype thereby decreasing fibrosis and VNH.

Periadventitial therapies for vascular surgery are predicated on the local application of biomaterials, small-molecule drugs, signaling molecules, gene therapies, and/or cells to the vascular adventitia (13). Compared to systemic or intraluminal delivery, periadventitial treatments target the vascular wall, reduce off-target effects and systemic toxicity, and avoid endothelial damage. They also offer the advantage of prophylactic, intra-operative application to encourage AVF maturation. We previously reported that systemic administration of the LOX inhibitor β-aminopropionitrile (BAPN) increased blood flow and decreased LOX activity determined by reduced collagen crosslinking following AVF creation in rats (3). BAPN binds the active site of LOX, preventing ECM crosslinking (14). In this study, we engineer a periadventitial nanofiber scaffold wrap for directional, controlled BAPN delivery into the AVF venous limb to improve vascular remodeling. We study the effects of periadventitial BAPN inhibition on AVF remodeling and fibrosis by studying the changes in ECM composition and cell phenotypes. We hypothesize that periadventitial BAPN treatment is a viable strategy to indirectly promote adaptive venous remodeling after AVF creation by reducing ECM stiffness, thereby perturbing fibrotic signaling, preserving functional vascular cell phenotypes, and promoting optimal vascular function.

## Materials and methods

### AVF creation in rats

Sprague Dawley rats (200–350 g) of both sexes were purchased from Envigo (Indianapolis, IN). AVFs were created by an end-to-side anastomosis of the epigastric vein to the nearby femoral artery as previously described (15). Briefly, the femoral artery and epigastric vein are dissected and clamped. A 1 mm arteriotomy is created in the side of the artery, and the distal end of the vein is anastomosed to the artery using 10.0 monofilament sutures. BAPN-loaded or vehicle scaffolds (5 mm x 5 mm) were wrapped around the juxta-anastomotic zone of the epigastric vein immediately after AVF creation (Supplementary Figure 1). The surgical success rate was 100%. The 21-day patency rate was 86.2 and 78.5% for the BAPN and vehicle groups, respectively. In total, 47 rats of both sexes underwent surgery for AVF creation and were randomly allocated for histology (*n* = 27), pressure myography (*n* = 12), or RNA sequencing (*n* = 8). Seven animals were excluded from the study because of absence of patency or not being suitable for histology. Flow was measured with a perivascular transit time flowmeter (Transonic Systems Inc., Ithaca, NY) at the time of AVF collection. Animals were euthanized with an overdose of isoflurane and AVF were harvested and stored in 10% formalin for histology or calcium-free physiological saline (PSS) for pressure myography. The Institutional Animal Care and Use Committee at the University of Miami approved all studies.

### Nanofiber fabrication and characterization

Bilayer nanofiber scaffolds were fabricated with a custom-made electrospinning apparatus (16) using a two-stage process (Supplementary Figure 2B). Ester-terminated poly (lactic-co-glycolic acid) [PLGA; (15% w/v, Resomer^®^ RG 504, Sigma-Aldrich)] and poly (–lactic acid) [PLA; (10% w/v, Poly(L-lactide) Sigma Aldrich)] were prepared by stirring in hexafluoroisopropanol overnight. For BAPN-loaded scaffolds, BAPN was added to the PLGA melt before stirring (10% w/w PLGA). Poly-lactic acid (PLA) (Sigma-Aldrich) solutions (10% w/v) were prepared in a similar manner. The perivascular PLA layer (2 mL) was spun first followed by the drug-loaded periadventitial PLGA layer. The PLA solution (2 mL) was loaded into a 5mL syringe with a 22-gauge blunt stainless-steel needle. A 20 kV current was applied to the needle, and the solution was pumped at 2 mL/h using a syringe pump (World Precision Instruments, Sarasota, FL) at a working distance of 15 cm. Nanofibers were collected on an aluminum-wrapped ground. Upon completion of the perivascular layer, the syringe was replaced with a syringe containing the PLGA melt (3 mL), and the voltage was increased to 22 kV. Monolayer controls were prepared in the same manner without the PLA layer. Scaffolds were left in a biosafety cabinet overnight to evaporate excess solvent before cutting into 5 mm × 5 mm pieces yielding scaffolds with 142.5 μg of BAPN/scaffold. Scaffolds were sterilized with ultraviolet light for 10 min. Fiber architecture and diameter was confirmed using scanning electron microscopy (SEM) (JEOL Ltd., Tokyo, Japan) after gold sputter-coating (Supplementary Figures 2C, D).

### BAPN release profile and directional release

PLGA scaffolds (5 mm × 5 mm) were weighed and placed in 1.0 mL of phosphate-buffered saline (PBS) and incubated at 37°C. PBS was collected and replaced with fresh PBS at specified timepoints. The collected PBS was stored at −20°C to determine cumulative BAPN release using high-performance liquid chromatography (HPLC).

For HPLC, a gradient flow (0.5 mL/min) of 60% H2O and 40% methanol was used as the eluent with a C18 column. BAPN peaks were observed at a retention time of 1.80 min using a UV detector (220 nm). The concentration of BAPN was calculated based on a standard calibration curve of the peak integrations. Percent release was calculated as the ratio of the mass of BAPN in the supernatant to the initial mass of BAPN in the PLGA scaffold.

Directional release was determined using a Franz Cell diffusion apparatus (PermeGear, Hellertown, PA). Methylene blue was used as a model compound due to its hydrophilicity and similar molecular weight. Bilayer scaffolds (1 cm × 1 cm) were flipped on either the perivascular (PLA) or periadventitial (PLGA) side and placed across the orifice of the receptor chamber before clamping the donor chamber. Both chambers were filled with PBS, and the PBS from the donor chamber was collected replaced at specified timepoints. The methylene blue concentration was measure using a plate reader at 560 nm. Monolayer scaffolds flipped on either side were used as controls.

### Histology and immunochemistry

After embedding in paraffin, AVFs were sectioned into 5 μms beginning at the anastomosis. Sections were either stained with an Elastin Van Gieson kit (Sigma Aldrich) or Masson’s Trichrome (Polysciences Inc., Warrington, PA) kits according to the manufacturer’s protocol to measure morphometry or fibrosis, respectively. For morphometry, circumferential vessel layers were traced using the elastic laminae as guides. First, the entire vessel cross section was traced and measured. The lumen area was determined by tracing the internal elastic lamina (IEL) and measuring the area. Total wall area (media + adventitia) was calculated by subtracting the lumen area from the cross-sectional area. Neointimal area was determined by tracing any tissue growth beyond the IEL into the lumen. Intimal Growth (I/I + W) was calculated from the ratio of the neointimal area and the combination of the neointimal and wall area. For trichrome quantification, images were deconvoluted into appropriate channels using the FIJI add-on. Percent area was calculated in the blue channel by applying the same threshold to all images.

For immunohistochemical staining, sections were rehydrated by serially immersing them in xylene, alcohol, and water. For cellular markers, antigen retrieval was performed by boiling slides in 10 mM citrate buffer (pH 6.0) for 20 min. ECM proteins were retrieved enzymatically by incubating in proteinase K (20 μg/ml) for 10 min. Sections were blocked for 10 min with Dako Peroxidase block followed with Dako Protein Block for 1 h (Agilent, Santa Clara, CA). AVFs were incubated with primary antibodies overnight at different dilutions. Negative controls were incubated with antibody diluent (DAKO) instead (Supplementary Figure 3). Bound antibodies were detected using the Dako Universal Link kit (Agilent, Santa Clara, CA), and color was developed with AEC Substrate Chromogen for 10 min (Abcam). Nuclei were counterstained with hematoxylin before mounting with aqueous mounting media. Images were acquired using a VisionTek DM01 digital microscope (Sakura Finetek, Torrance, CA). Percentage of positive area was determined using FIJI color deconvolution. For adventitia versus media measurements, a trained, blinded independent observer traced the media or adventitia before calculating the percent area.

### Doppler ultrasound

Doppler ultrasound (Vevo Visualsonic 3100, Fujifilm) was used to serially monitor AVF patency and dilation in animals being used for either histology or pressure myography. Briefly, anesthesia was induced using 5% isoflurane and maintained at 2%. Animals were placed on the platform in supine position and the hair near the epigastric vein was removed. Ultrasound was performed using the MX100 rat probe. The AVF was imaged in C-Mode using a PRF of 5–10 kV. Flow was also recorded in the feeding artery and AVF using the pulse-wave velocity feature. Vessel diameter was measured in the VevoLab software by drawing six lines across the vessel lumen beginning at the anastomosis. The average length was used as the AVF diameter. Percent dilation was determined by comparing the AVF diameter to the epigastric vein.

### Pressure myography

Arteriovenous fistulas were cleaned of fat so the wall could be clearly distinguished. AVFs were then mounted onto the pressure myography apparatus (DMT Model 110P, Danish Myo Technology, Ann Arbor, MI) by securing the proximal and distal segment of the AVF on a pair of steel cannulas (outer diameter: 1200 μm) with surgical nylon sutures. After the distal segment of the vessel was secured, and the vessel length adjusted to eliminate stretch, the intraluminal pressure was raised to 100 mmHg to equilibrate the vessels. The mounted vessels were equilibrated for 30 min at 37°C in calcium-free PSS gassed with a mixture of 95% O2 and 5% CO2, before reducing the intraluminal pressure to 5 mmHg. To obtain the pressure-diameter curve, the internal and external diameters of the AVF, distal to the scaffold, were measured while increasing intraluminal pressure in 5 mmHg increments between 10 and 100 mmHg. The diameters were recorded using the DMT MyoView 4 software (Danish Myo Technology). Distensibility was calculated as % change in diameter at increasing pressures with respect to the diameter at 10 mmHg. The circumferential wall strain and stress were first determined to calculate the incremental elastic modulus (Einc) as previously described (17).

### Bulk RNA sequencing

RNA was extracted using Trizol and treated for DNAse before submitting samples to the University of Miami John P. Hussman Institute for Human Genomics, Center for Genome Technology. Total RNA was quantified and qualified using the Agilent Bioanalyzer to have an RNA integrity score > 5. Then 500 ng of total RNA was used as input for the Illumina TruSeq Stranded Total RNA Library Prep Kit with Ribo-Zero to create ribosomal RNA–depleted sequencing libraries. Each sample was sequenced to more than 40 million raw reads on the Illumina NextSeq500. Raw counts were normalized by Log_2_ fold change using the Deseq plug-in in R Studio. Genes with a base mean < 100 were excluded from analysis. Volcano plots were constructed using GraphPad Prism. Gene Ontology Pathway Analysis was used to identify differentially expressed pathways. The heat map and dot plot were generated in R studio.

### Statistical analysis

Statistical analyses were performed using GraphPad Prism version 9 for Windows (GraphPad Software, San Diego, CA). Normally distributed data were compared using unpaired *t*-tests with Welch’s correction and expressed as mean ± SD. A *p*-value < 0.05 was considered significant.

## Results

### Bilayer nanofiber scaffolds exhibit sustained and directional release

**FIGURE 1:**
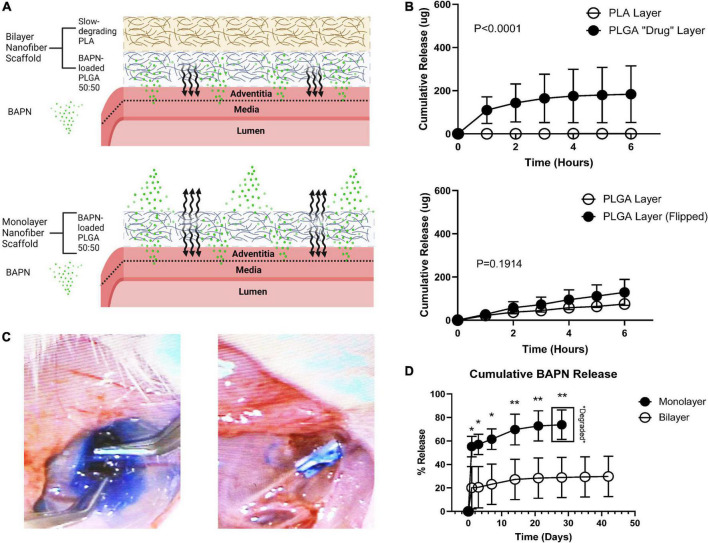
Periadventitial bilayer nanofibers confer controlled BAPN release. **(A)** Bilayer wraps (top) were fabricated using a slow-degrading PLA perivascular layer followed by a drug-loaded periadventitial PLGA layer to confer directional BAPN delivery into the venous adventitia. Made with Biorender.com **(B)** release of methylene blue dye from the perivascular and periadventitial interfaces (*n* = 3) of bilayer and monolayer nanofiber wraps in a Franz Diffusion Cell. Bilayer wraps only released dye from the periadventitial PLGA “drug” layer (*p* < 0.001) (top) while there was no difference in release between the perivascular and periadventitial interfaces in monolayer wraps (*p* = 0.1914) (bottom). **(C)** Release of methylene blue from monolayer (left) and bilayer (right) nanofiber scaffolds *in vivo*. **(D)** Cumulative release of BAPN from monolayer and bilayer scaffolds. Monolayer nanofiber scaffolds had a tremendous burst release and were completely degraded at day 28. Bilayer scaffolds sustained release over 6 weeks (Each dot represents four replicates. *p < 0.05, **p < 0.01, unpaired *t*-test).

Periadventitial biomaterials localize therapeutics to the vascular wall following AVF creation. Directional delivery has emerged as a prerequisite feature for periadventitial biomaterials to maximize their therapeutic effects as it ensures high concentrations within the vascular wall, prevents therapeutic loss into the perivascular space, and thereby reduces off-target effects (18). We electrospun nanofibrous BAPN-loaded scaffolds that can be placed directly over the venous limb following AVF creation, supporting the vein and providing sustained BAPN release. These scaffolds were fabricated to have a two-layer design (bilayer) comprised of an outer slow-degrading PLA layer and inner adventitia-contacting BAPN-loaded PLGA layer to facilitate directional release compared to our previous design (monolayer) (3) (Figure 1A). To confirm directional release, we utilized a Franz Diffusion chamber which is commonly used to study the diffusion of molecules through membranes, allowing us to quantify the amount of drug being released from the inner and outer surfaces of our scaffolds. Importantly, the bilayer design only released its load from the adventitia-contacting “drug layer” while the monolayer design had similar release from both the perivascular and periadventitial interfaces (Figures 1B, C). Bilayer scaffolds attenuated the initial burst release seen in monolayer scaffolds, releasing only 20% of its load in the first 3 days compared to almost 60% with the monolayer (*p* < 0.05) (Figure 1D). Also, the bilayer design had a slower release profile, extending BAPN release over 6 weeks while the monolayer scaffolds were completely degraded by 1 month.

### Periadventitial BAPN delivery improves AVF outward remodeling

**FIGURE 2:**
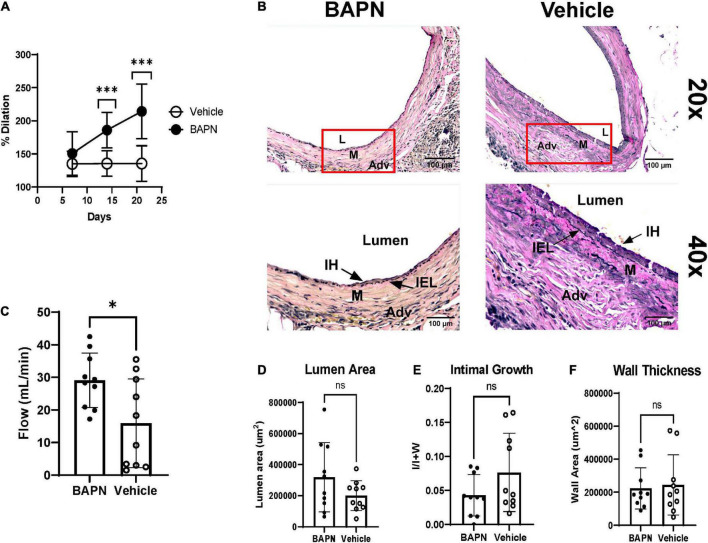
Dilation, flow, and histomorphometry of AVFs after 21 days. **(A)** Lumen dilation of AVFs over 21 days. (Top) BAPN treatment led to a significant increase in dilation at days 14, and 21 compared to vehicle controls (Each dot is ten replicates. ^***^p < 0.005, unpaired *t*-test). **(B)** Representative images of AVFs stained with Elastin Van Gieson at day 21. Top images are at 20× magnification. Bottom images are 40× magnification. Scale bar = 100 μm (Adv, adventitia; M, media; IH, intimal hyperplasia; L, lumen). **(C)** Venous flow at 21 days. BAPN significantly increased AVF flow (29.08 ml/min vs. 15.94 ml/min; *n* = 10, *p < 0.05, unpaired *t*-test). **(D–F)** Lumen area (*p* = 0.1474) **(D)**, venous intimal hyperplasia (*p* = 0.1254) **(E)**, and wall area (ns) **(F)** were determined by tracing the lumen, internal elastic lamina, media, and adventitia.

β-aminopropionitrile and vehicle bilayer scaffolds were wrapped around the juxta-anastomotic zone of the outflow vein immediately after AVF creation. We employed ultrasound to monitor patency and dilation throughout the time of the study. All surgeries were successful, and animals remained alive until euthanasia. The BAPN group had increased lumen dilation compared to vehicle controls at day 14 (185.9% vs. 135.5%; *p* < 0.005) and day 21 (214.2% vs. 135.5%; *p* < 0.005) (Figure 2A). The BAPN-treated group continued to dilate over 3 weeks while vehicle-treated AVFs reached peak dilation at day 7. To further investigate the effects of treatment on AVF remodeling, we conducted histomorphometric analysis using EVG staining to delineate the vessel layers. Both BAPN and vehicle scaffolds led to AVFs with open lumens (Figure 2B). However, BAPN increased flow in outflow veins at the time of sacrifice (29.08 ml/min vs. 15.94 ml/min; *p* < 0.05) (Figure 2C). Treatment with BAPN scaffolds showed a trend toward reduced VNH compared to vehicle controls while there was no difference in lumen or wall area (Figures 2D–F). However, there was a significant difference in lumen area between AVFs treated with BAPN scaffolds and AVFs without any intervention (Supplementary Figure 4). In all, these data show that periadventitial LOX inhibition improves AVF remodeling yielding a high flow, dilated access without jeopardizing wall remodeling.

### Periadventitial LOX inhibition reduces postoperative fibrosis and improves compliance

**FIGURE 3:**
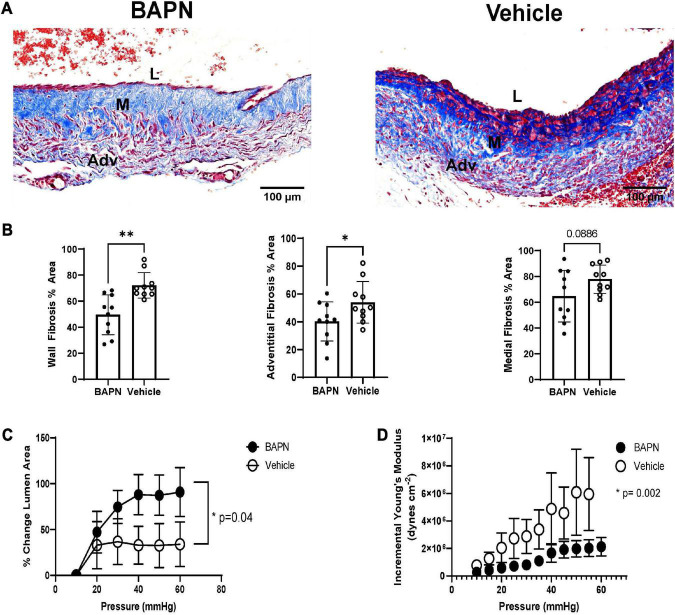
AVF fibrosis and compliance after 21 days. **(A)** Masson’s Trichrome staining of BAPN and vehicle AVFs. Scale bar = 100 μm (Adv, adventitia; M, media; L, lumen). **(B)** BAPN significantly reduced trichrome staining throughout the entire wall and the adventitia while the difference between medial collagen content did not reach significance at 21 days (*n* = 10; *p < 0.05, ^**^p < 0.01, unpaired *t*-test). Pressure myography showed BAPN treatment increased distensibility **(C)** (*p* < 0.05, unpaired *t*-test) while reducing incremental young’s modulus (*p* < 0.005, unpaired *t*-test) **(D)**.

After demonstrating the impact of bilayer BAPN nanofiber scaffolds on AVF outward remodeling, we measured their impact on postoperative fibrosis at 21 days. BAPN-treated AVFs had a significant reduction in fibrosis in the entire wall (*p* < 0.01) (Figures 3A, B). To further understand the impact of periadventitial LOX inhibition with bilayer BAPN nanofiber scaffolds, we measured fibrosis per vessel layer. BAPN scaffolds specifically reduced fibrosis in the adventitia layer (*p* < 0.05) and not the medial layer (Figure 3B). To determine the functional impact of reduced fibrosis, we measured AVF compliance using pressure myography. BAPN-treated AVFs had a significant increase in distensibility (Figure 3C) which was associated with reduced Einc, a pressure-dependent metric of vascular stiffness (Figure 3D), indicating that periadventitial BAPN treatment is effective at preventing fibrotic stiffening and promoting venous compliance.

### Periadventitial LOX inhibition does not alter fibrous ECM composition

**FIGURE 4:**
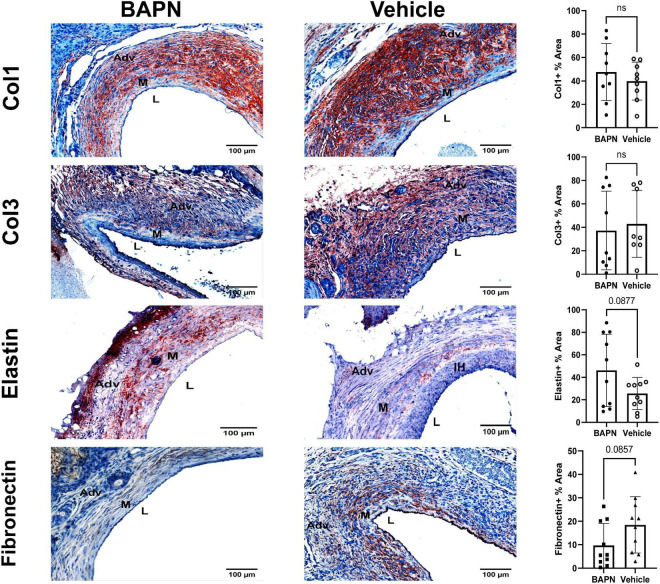
Effects of BAPN-releasing nanofibers on ECM composition. **(First Row)** representative Col1 immunohistochemistry images of BAPN and vehicle AVFs on day 21. There was no difference in Col1 % area at day 21. **(Second Row)** Representative Col3 immunohistochemistry images of BAPN and vehicle AVFs on day 21. There was no difference in Col3 % area at day 21. **(Third Row)** Representative elastin immunohistochemistry images of BAPN and vehicle at day 21. Elastin % area showed an increasing trend on day 21. **(Fourth Row)** Representative fibronectin immunohistochemistry images of BAPN and vehicle at day 21. Fibronectin % area trended toward reduction in the BAPN group. Scale bar = 100 μm (Adv, adventitia; M, media; L, lumen; IH, intimal hyperplasia).

The ECM plays a significant role in tissue biomechanics and cell differentiation (4, 19). It has also been shown to play an important role in AVF maturation in mice and humans (20, 21). LOX activity controls ECM gene expression and therefore may affect the ECM composition (22). Accordingly, we used immunohistochemistry to quantify key ECM proteins involved in vascular remodeling. Fibronectin, a scaffolding protein involved in collagen deposition and cell-ECM interactions, trended toward significant reduction at day 21 for BAPN treated AVFs while there was no difference in the amounts of collagen I and collagen III at day 21 (Figure 4). Elastin also trended toward a significant increase in the BAPN group at 21 days (*p* = 0.09). In all, these findings suggest that periadventitial LOX inhibition has little impact on the overall composition of the ECM and changes in tissue biomechanics result from a change in ECM crosslinking and organization rather than composition.

### Periadventitial LOX inhibition modulates myofibroblast differentiation and reduces profibrotic markers

**FIGURE 5:**
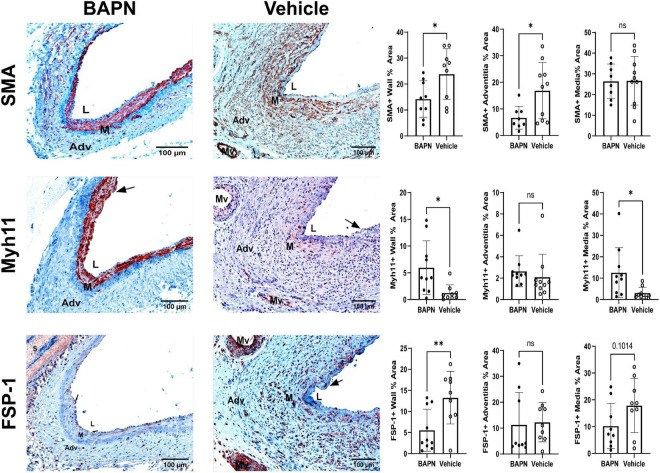
Periadventitial lOX inhibition promotes a contractile SMC phenotype. **(First Row)** Representative SMA immunohistochemistry images of BAPN **(left)** and vehicle **(right)** AVFs on day 21. SMA + % area was significantly reduced in the entire wall and adventitia at day 21 (*p < 0.05, unpaired *t*-test). **(Second Row)** Representative MYH11 immunohistochemistry images of BAPN **(left)** and vehicle **(right)** AVFs on day 21. BAPN AVFs had increased MYH11 + staining in the entire wall and media at day 21 (*p < 0.05, unpaired *t*-test). **(Third Row)** Representative FSP-1 immunohistochemistry images of BAPN **(left)** and vehicle **(right)** AVFs on day 21. FSP-1+ % area was significantly reduced in the BAPN group at day 21 (^**^p < 0.01). Scale bar = 100 μm (Adv, adventitia; M, media; Mv, microvessel; L, lumen, arrows show intimal hyperplasia).

To assess the effects of LOX inhibition on the ECM with changes in cellular phenotypes, we performed a series of stains to distinguish VSMC and MF phenotypes. By day 21, there was a significant reduction of SMA in the BAPN group (Figure 5). The BAPN group had primarily medial SMA expression while there was a high number of SMA + cells in the adventitia of the vehicle group at day 21. Myh11 expression tended to align with SMA in the BAPN group and was significantly increased at day 21. Further analysis showed there was an increase in medial Myh11, indicating that the treatment was protective of contractile VSMC. BAPN-treated AVFs had a significant decrease in FSP-1 which could be observed throughout the vessel wall. In all, these results suggest that periadventitial LOX inhibition using BAPN-loaded nanofibers attenuates cell differentiation into MFs and promotes a contractile VSMC phenotype.

**FIGURE 6:**
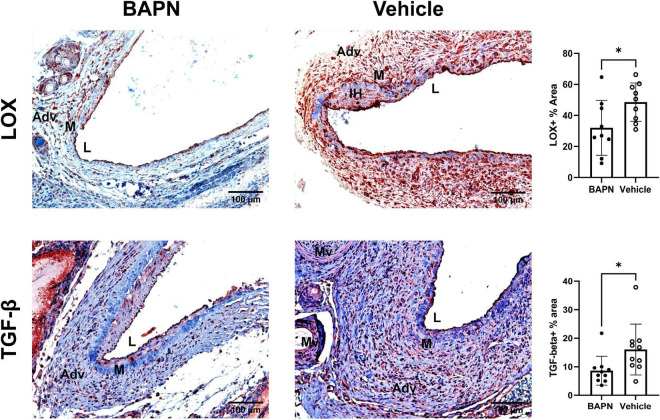
Periadventitial LOX inhibition reduces profibrotic markers. **(Top Row)** Representative LOX immunochemistry images of BAPN **(left)** and vehicle **(right)** AVFs on day 21. LOX+ % area was significantly reduced in the BAPN group by day 21 (*p < 0.05, unpaired *t*-test, *n* = 9). **(Bottom Row)** Representative TGF-β immunohistochemistry images of BAPN **(left)** and vehicle **(right)** AVFs on day 21. TGF-β % area was significantly reduced at day 21 (*p < 0.05, unpaired *t*-test). Scale bar = 100 μm (Adv, adventitia; M, media; Mv, microvessel; L, lumen).

Fibrosis is characterized by increased LOX and TGF-β which are both associated with AVF non-maturation (3, 23). Being that MFs are known to also express LOX (24), we checked for differences in LOX expression due to LOX inhibition with BAPN scaffolds. LOX expression was significantly reduced by day 21 in the BAPN group (Figure 6) while LOX + cells populated the entire wall including the neointima in the vehicle group. TGF-β is known to induce MF differentiation, leading to fibrosis (25). TGF-β expression was significantly reduced at day 21 in the BAPN group. These results indicate that periadventitial LOX inhibition may reduce fibrosis by attenuating expression of LOX and TGF-β.

### BAPN alters matrisome gene expression

**FIGURE 7:**
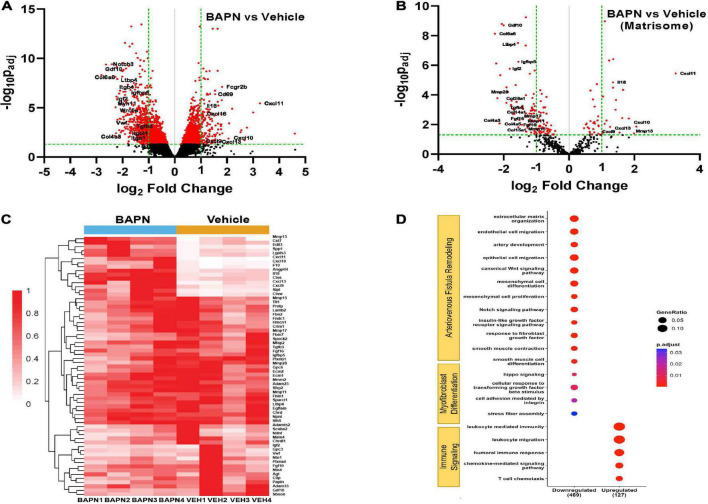
Differential AVF gene expression with BAPN treatment. Volcano plot of all genes **(A)** and matrisome genes **(B)** with BAPN Treatment at 21 days. Red dots = p_*adj*_ < 0.05. **(C)** Heat map of matrisome genes upregulated log_2_ fold > 1.0 or downregulated log_2_ fold < –1.0 compared to vehicle controls. **(D)** Gene ontology pathway analysis of differentially expressed p_*adj*_ < 0.05 matrisome genes. Pathways associated with AVF remodeling and myofibroblast differentiation were downregulated while pathways related to immune signaling were upregulated with BAPN treatment.

To further understand the effects of periadventitial LOX inhibition using BAPN on AVF remodeling, we performed bulk RNA sequencing on outflow veins from rats treated with BAPN and vehicle scaffolds. The volcano plot shows there was a total of 2629 differentially expressed genes (FDR < 0.05). Of these, there were 157 genes that were upregulated log2 fold > 1.0 and 532 genes that were downregulated log2 fold < −1.0 (Figure 7A). Further analysis revealed differential expression of matrisome genes (Figures 7B, C), suggesting BAPN affects AVF remodeling by perturbing the ECM structure and related signaling pathways. The LOX substrates *Cola1* and *Col3a1* were both downregulated, but not less than log2 fold < −1.0 (log2 fold −0.919 and log2 fold −0.806, respectively). However, *Col6a6*, *Col28a1*, *Col14a1*, *Col4a3*, *Col4a5*, and *Col15a1* were all strongly downregulated. The TGF-β gene, *Tgfb1* (log2 fold −0.836) and its receptors *Tgfbr2* (log2 fold −0.722) and *Tgfbr3* (log2 fold −0.918) were significantly downregulated (FDR < 0.05). In addition, secreted factors *Gdf10*, *Igf2*, *Tgfb3*, *Fgf16*, and *Fgf10* were substantially downregulated in addition to the ECM complexes latent transforming growth factor binding protein (LTBP) *Ltbp4* and insulin growth factor binding protein *Igfbp5* (log2 fold < −1.0, FDR < 0.05). Alternatively, BAPN significantly enriched genes pertaining to immune signaling, especially immune cell migration, including members of the CXC family *Cxcl9*, *Cxcl10*, *Cxcl11*, *Cxcl13*, and *Cxcl16* and Il18 suggesting BAPN enables improved immune trafficking and T cell chemotaxis. The matrix metalloprotease (MMP) family plays a crucial role in ECM remodeling, especially after AVF creation. The collagenase *Mmp13* was strongly upregulated while *Mmp11*, *Mmp15*, *Mmp17* and *Mmp28* were downregulated, indicating BAPN exerts a pleiotropic effect on ECM matrix degradation. Gene ontology pathway analysis showed BAPN downregulated pathways associated with Arteriovenous Fistula remodeling and Myofibroblast Differentiation including cellular response to TGF-β signaling at 21 days while pathways associated with Immune Signaling, especially the adaptive immune response, were upregulated (Figure 7D).

## Discussion

In the present study, we have demonstrated that periadventitial LOX inhibition using biodegradable PLGA nanofiber scaffolds with directional BAPN release improves AVF vascular remodeling in rats. We successfully fabricated bilayer nanofibrous scaffolds which sustained BAPN release and conferred directional release behavior compared to single-layer scaffolds. We performed a combination of functional and histological analyses to show that periadventitial LOX inhibition is a feasible strategy to promote outward remodeling, prevent fibrosis, and improve biomechanical properties in AVF *in vivo*. BAPN treatment with bilayer scaffolds improved vein diameter, flow, vascular compliance and reduced adventitial fibrosis. Analysis of cell phenotypes revealed BAPN treatment decreased SMA +, FSP-1 +, and LOX + cells and increased Myh11 + cells at day 21 which was associated with a decrease in the profibrotic TGF-β staining.

Researchers have sought periadventitial therapies to improve AVF outcomes for decades (26–28). We previously demonstrated the feasibility of pan-LOX inhibition using BAPN as a promising strategy to encourage adaptive vascular remodeling when delivered systemically in rat AVFs (3). The rat AVF model is preferable to mouse models as it uses larger, peripheral vessels, resembling that of clinical AVF. It also recapitulates the features of vascular remodeling observed in humans and porcine models such as upregulation of matrix metalloprotease (MMP)-2 and – 9 and the formation of venous stenosis and a VNH lesion comprised of SMA + cells (29). In this earlier study, we used an electrospun nanofiber wrap as a proof-of concept to compare local delivery with systemic administration. We showed that local LOX inhibition reduced fibrosis and collagen crosslinking and improved AVF distensibility and stiffness compared to vehicle controls. In the current study, we optimize our scaffold, utilizing a bilayer design to control the direction of BAPN release thereby preventing perivascular drug loss and improving release characteristics to maximize its efficacy. With this design, we demonstrate a significant improvement in blood flow compared to the previous study. In addition, we analyze the effects of sustained, directional LOX inhibition on the biological aspects of vascular remodeling at the tissue, cellular, and genetic level that were not assessed in previous studies.

A plethora of strategies have been proposed to promote outward vein remodeling after AVF creation. Topical recombinant elastase has been proposed to improve AVF dilation and maturation by cleaving elastic fibers to allow for luminal expansion (30). Alternatively, BAPN may be used to encourage outward remodeling, especially within the AVF proteolytic microenvironment (31). However, a local, controlled delivery system is required to promote dilation without aneurysms or lathyrogenic effects (32). Herein, we describe the feasibility of a bilayer PLGA-based nanofiber scaffold for the unidirectional, sustained periadventitial delivery of BAPN to newly created AVFs, maximizing the therapeutic effect of BAPN and providing mechanical support to the vessel during remodeling similar to an external stent (33). PLGA has been employed as a biomaterial for drug delivery due to its biocompatibility and tunable release properties (34) while nanofibers are commonly employed in tissue engineering as scaffolds due to their biodegradability, porosity, ECM-like architecture which enable cell-mediated remodeling and tissue integration (35, 36). By combining PLGA nanofiber layers with different degradation, and consequent release rates, it was possible to control the direction of drug release (37). It is also essential to maintain therapeutic BAPN levels to achieve an effect, as BAPN-depleted LOX is quicky replenished (38). Our nanofiber system provided controlled BAPN release for more than a month, the typical timeframe for maturation, while attenuating the burst release seen with our previous single-layer design. Indeed, periadventitial LOX inhibition with BAPN-loaded nanofibers improved AVF dilation over 3 weeks and led to adaptive remodeling using a much smaller dose (142.5 μg) than aneurysm and dissection models which typically co-administer BAPN with angiotensin II or elastase (39).

There has been copious research into LOX inhibition as a potential strategy to prevent fibrosis and pathological microenvironments (40, 41). BAPN functions to irreversibly inhibit the activity of LOX and its analogs by binding to the active site, preventing the post-translational crosslinking of collagen and elastin fibrils and reducing tissue stiffness. Indeed, we show that periadventitial LOX inhibition with BAPN-releasing scaffolds decreased fibrosis, especially in the adventitia which has been previously associated with AVF failure (42), and improved flow and vascular compliance. Interestingly, LOX inhibition had little effect on the amount of the fibrous collagens within the vascular wall despite reducing fibrosis. It has been reported that increased tissue stiffness results from dysregulated LOX activity and subsequent changes in ECM architecture rather than increases in collagen concentration and that LOX inhibition is a viable strategy to improve tissue function and normalize collagen fibrillogenesis (43).

MFs have been implicated in vascular stenosis, fibrosis and VNH (9, 10). MFs arise from mesenchymal cells that acquire expression of SMA, activated adventitial fibroblasts or synthetic VSMCs that lose contractile gene expression. Periadventitial BAPN scaffolds reduced SMA + and FSP-1 + expression, indicating a decrease in MFs. Additionally, BAPN increased MYH11 + staining indicating an enhancement in differentiated VSMCs. Both VSMCs and adventitial fibroblasts alter their phenotypes in response to stiffness *via* TGF-β signaling (44, 45). TGF-β and LOX are both implicated in AVF failure and MF differentiation (3, 46) and were reduced with BAPN. LOX expression is induced by TGF-β (47) so the reduction in both factors may be intricately related. While cell lineages were not determined, these results reveal periadventitial BAPN therapy encourages favorable AVF remodeling by perturbing the fibrotic feedback loop (24, 48) and preserving a differentiated VSMC phenotype.

LOX primarily exerts its effects through the posttranslational crosslinking of ECM proteins but also regulates the promoter activity of its substrates collagen III and elastin (49, 50), meaning LOX inhibition may affect fibrosis *via* multiple mechanisms. Accordingly, we utilized bulk RNA sequencing and subsequent pathway analysis to deconvolute the effects of periadventitial LOX inhibition on AVF remodeling and fibrosis. Surely BAPN had a drastic effect on the expression of matrisome genes, indicating its benefits stem from the impact of LOX inhibition on ECM structure. We hypothesized BAPN would impact the deposition of the fibrous collagens collagen I and collagen III yet noted no difference using immunohistochemistry, despite reduced trichrome staining. While both *Cola1* and *Col3a1* were downregulated with BAPN, there was abundant gene transcription in both groups (Supplementary Table 1), meaning differences at protein expressions might not be detectable using immunohistochemistry and a more direct method should be used. Still, this indicates that BAPN-treated AVFs had reduced fibrous collagen production while controls continued fibrotic deposition. Conversely, *Col4a3*, *Col4a5*, *Col6a6*, *Col14a1*, *Col15a1*, and *Col28a1* were all substantially downregulated in the BAPN group suggesting LOX inhibition may hinder fibrosis by influencing basement membrane and microfibrillar protein synthesis and explains the reduction in trichrome staining despite no reduction in collagen I and III staining.

Pathways associated with AVF remodeling were primarily downregulated with BAPN treatment after 21 days, including cell proliferation, migration, differentiation, and ECM organization. Of interest, Smooth muscle cell differentiation, and Smooth muscle contraction were both downregulated; one possible explanation is that AVFs treated with BAPN had completed their remodeling program by 21 days so genetic transcripts had achieved steady state. Indeed, BAPN treated AVFs already had significant lumen expansion and arterialization as evidenced by histology. For example, *Myh11* was significantly reduced at the RNA level despite increased MYH11 + staining.

TGF-β, BMP, and insulin growth factor are all associated with AVF stenosis (23, 51–53). TGF-β in particular plays an important role in AVF vascular remodeling, regulating ECM deposition and VSMC proliferation (54, 55). *Gdf10, Tgfb3*, and *Igf2* were strongly downregulated with BAPN treatment along with TGF-β and insulin growth factor ECM binding proteins, *Ltbp3*, *Ltbp4* and *Igfbp5*. TGF-β family members are secreted as part of a large latent complex (LLC) that consists of TGF-β, a prodomain known as the latency associated peptide (LAP), and LTBPs which allow for storage in the ECM (56). ECM contraction by SMA expressing cells releases TGF-β from the LLC, allowing it to activate its receptor and in turn enhancing the expression of α-SMA and subsequent MF differentiation (57). Reduced tissue compliance has previously been shown to reduce MF differentiation by preventing the release of TGF-β from the LLC (45). In this study, we show that periadventitial LOX inhibition both reduced stiffness and SMA expression in AVF veins which was associated with a downregulation of genetic processes related to MF differentiation (Figure 7D). Indeed, pathway analysis revealed a reduction in cell-matrix adhesion and cell adhesion mediated by integrin which was associated with significant differential expression of a slew of integrin receptors including *Itga2b, Itgb4, Itgbl1, Itga7, Itga1* with BAPN treatment. Integrin binding functions in MF differentiation *via* two mechanisms: besides cell-ECM interactions that affect cytokine sequestration, integrin binding is directly involved in MF differentiation and fibrosis through mechanosensitive pathways such as Hippo-YAP/TAZ signaling (25). *Yap1* and *Hippo signaling* were decreased in the BAPN group. It is possible LOX inhibition indirectly impacts TGF-β signaling by reducing stiffness and rearranging cryptic ECM integrins while directly perturbing the mechanosensitive MF differentiation feedback loop that culminates with tissue fibrosis (48) and contributes to VNH and constrictive remodeling in AVF.

Lastly, we observed an upregulation of genes associated with the humoral immune response, especially T cell migration. The positive role of lymphocyte mediated processes on AVF function has been previously reported (58). We propose BAPN improves immune cell migration through its effect on ECM organization which in turn leads to improved T cell signaling, however, the mechanism by which this occurs must be elucidated. In all, findings from RNA sequencing indicate the effects of BAPN on AVF remodeling are intricate and multifaceted.

Still, this study has its limitations. Since BAPN inhibits the entire LOX family, we are unable to determine inhibition of which LOX analogs mediates the therapeutic effect. Another shortcoming is that this report does not include any analysis on the organization of collagen and elastin in the ECM. Lastly, more research must be conducted to understand the mechanism by which reduced AVF stiffness translates into diminished MF differentiation.

In summary, this study describes a periadventitial nanofibrous scaffold that delivers BAPN directly to the AVF adventitia. This intervention was effective in preventing postoperative fibrosis and constrictive remodeling. In total, this data suggests sustained LOX inhibition using periadventitial nanofibrous scaffolds is a feasible approach to improve AVF maturation by encouraging outward remodeling and preserving compliance.

## Data availability statement

The raw data supporting the conclusions of this article will be made available by the authors upon request. The raw RNA-seq data is accessible in NCBI Gene Expression Omnibus through the GEO accession number: GSE225412.

## Ethics statement

This animal study was reviewed and approved by the Institutional Animal Care and Use Committee at the University of Miami.

## Author contributions

BA, FA, LM, and RV-P contributed to the idea and study design. BA fabricated and characterized the nanofiber scaffolds and performed all the remaining experiments and analysis. YW performed the AVF surgeries. BA and AG performed the histological staining and data analysis. MR performed the analysis for the RNA sequencing and constructed the respective figures. BA drafted the manuscript, which was critically reviewed and edited by RV-P, FA, and LM. RV-P and FA were responsible for supervision and mentorship. LM and XY contributed to the fruitful discussions and clinical insights. All authors took part in the interpretation of the results and approved the final version of the manuscript.

## Supplementary material

Supplementary Figure 1 Rat femoral-epigastric arteriovenous fistula model. **(A)** The end of the epigastric vein is anastomosed to the femoral artery. **(B)** Kaplan–Meier survival of AVF patency.

Supplementary Figure 2 Electrospinning of nanofiber scaffolds. **(A)** Diagram of electrospinning setup. Made with Biorender.com. **(B)** Picture of our custom electrospinning apparatus. **(C)** Scanning electron micrograph of bilayer PLA-PLGA nanofiber scaffolds. **(D)** Average fiber diameter of the PLA and PLGA layers.

Supplementary Figure 3 Antibodies and dilutions.

Supplementary Figure 4 Histomorphometry with Sham AVF controls.
