# Supplementary material for: Periadventitial β-aminopropionitrile-loaded nanofibers reduce fibrosis and improve arteriovenous fistula remodeling in rats
Source: Front Cardiovasc Med. 2023 Feb 28;10:1124106. doi: 10.3389/fcvm.2023.1124106 (PMC10011136; doi:10.3389/fcvm.2023.1124106)
Supplement: Supplementary file 1 [file Table_1.DOCX]

| **Gene** | **Base Mean** | **Log_2_ Fold Change** | **FDR** |
| --- | --- | --- | --- |
| *Col14a1* | 26541.71 | -1.23498 | 0.00261621 |
| *Col15a1* | 1042.132197 | -1.273166734 | 0.017008205 |
| *Col1a1* | 642383.1 | -0.91861 | 0.013338 |
| *Col28a1* | 111.9337243 | -1.756095942 | 0.00024705 |
| *Col3a1* | 617311.9 | -0.80555 | 0.015075 |
| *Col4a3* | 114.0162 | -2.12548 | 0.008477 |
| *Col4a5* | 1768.515 | -1.4388 | 0.010432 |
| *Col6a6* | 918.9906 | -2.27055 | 7.42E-09 |
| *Cxcl10* | 1360.465 | 2.060973 | 0.013643 |
| *Cxcl11* | 213.6827 | 3.263555 | 3.59E-06 |
| *Cxcl13* | 11755.05295 | 1.546296 | 0.035118 |
| *Cxcl9* | 1147.361711 | 1.02211187 | 0.041862198 |
| *Fgf10* | 187.2465941 | -1.246678076 | 0.011865157 |
| *Fgf16* | 194.2345599 | -1.172525409 | 0.006360908 |
| *Fgfr2* | 477.4320621 | -1.204580248 | 0.034211091 |
| *Gdf10* | 1043.429928 | -2.003294216 | 2.02E-09 |
| *Igf2* | 570.7135963 | -1.814722388 | 1.70E-06 |
| *Igfbp5* | 17619.09197 | -1.54951 | 5.83E-07 |
| *Itga1* | 1397.431808 | -1.216139359 | 0.037880706 |
| *Itga2b* | 222.1949252 | -1.145413043 | 0.008677228 |
| *Itga7* | 1203.835609 | -0.993182139 | 0.022861234 |
| *Itgb4* | 3294.780872 | -1.59740824 | 8.00E-08 |
| *Itgbl1* | 7015.276142 | -1.054532356 | 0.007572456 |
| *Ltbp3* | 7784.803614 | -0.935533688 | 0.005251236 |
| *Ltbp4* | 7477.758186 | -1.563806042 | 3.16E-08 |
| *Mmp11* | 173.2430243 | -1.12486 | 0.005271 |
| *Mmp13* | 828.8484986 | 1.99006041 | 0.039116809 |
| *Mmp15* | 523.2924704 | -1.007793484 | 0.018250544 |
| *Mmp17* | 325.0963077 | -1.316822319 | 0.003017413 |
| *Mmp28* | 229.0714019 | -1.875547356 | 6.39E-05 |
| Myh11 | 26253.92125 | -1.586125289 | 5.21E-06 |
| *Tgfb1i1* | 3136.922913 | -0.835723337 | 8.50E-05 |
| *Tgfb3* | 6628.595251 | -1.404716025 | 0.000906171 |
| *Tgfbr2* | 10602.35219 | -0.722448665 | 0.009982578 |
| *Tgfbr3* | 5129.655609 | -0.917724743 | 0.000967316 |
| *Yap1* | 1119.08588 | -1.200887006 | 0.003928349 |

Supplementary Table 1: Bulk RNA Seq Genes of Interest
