# Supplementary figures and images for: Periadventitial β-aminopropionitrile-loaded nanofibers reduce fibrosis and improve arteriovenous fistula remodeling in rats
Source: Front Cardiovasc Med. 2023 Feb 28;10:1124106. doi: 10.3389/fcvm.2023.1124106 (PMC10011136; doi:10.3389/fcvm.2023.1124106)

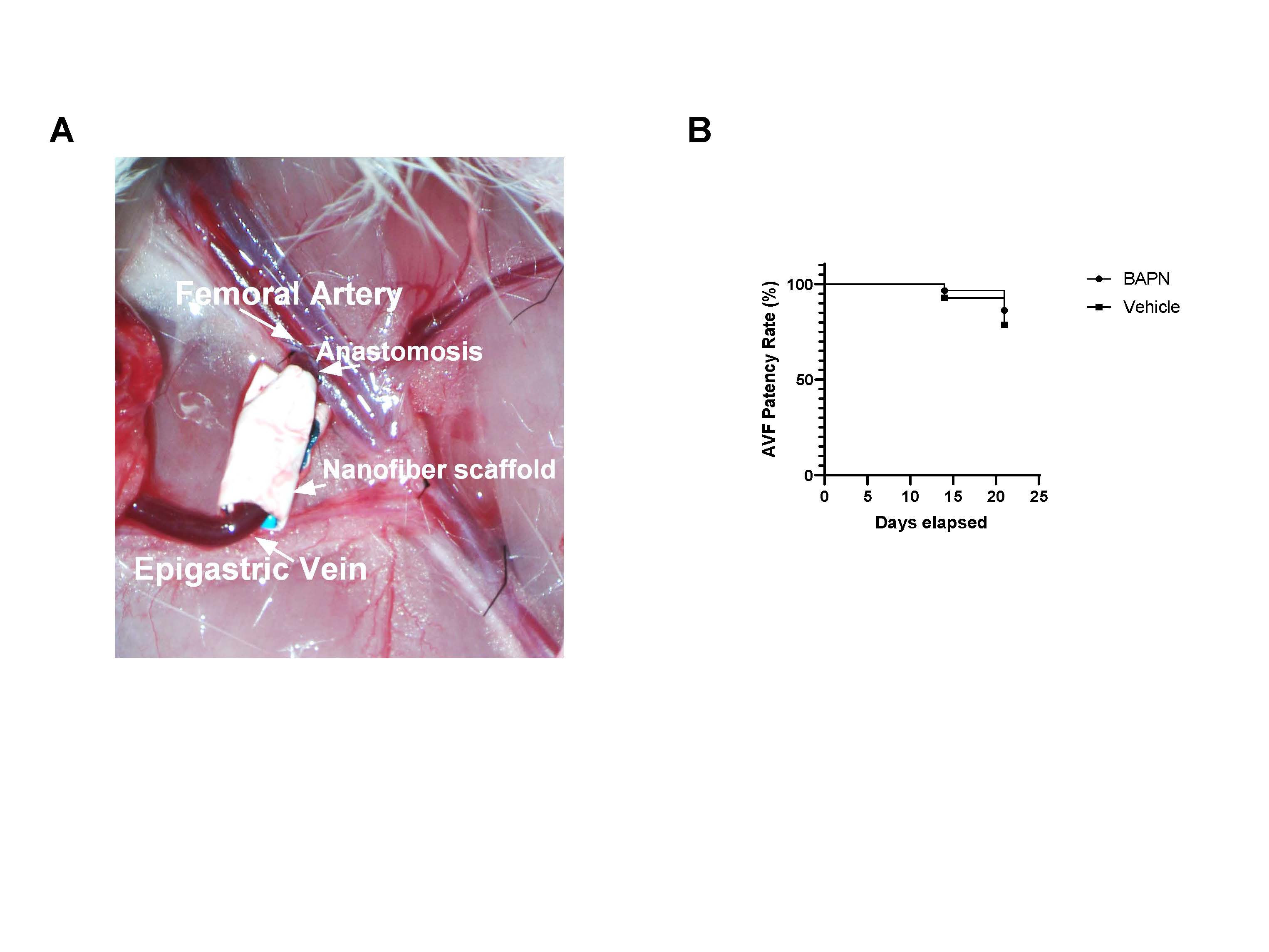

Supplement: Supplementary Figure 1 — Rat femoral-epigastric arteriovenous fistula model. (A) The end of the epigastric vein is anastomosed to the femoral artery. (B) Kaplan–Meier survival of AVF patency. [file Image_1.JPEG]

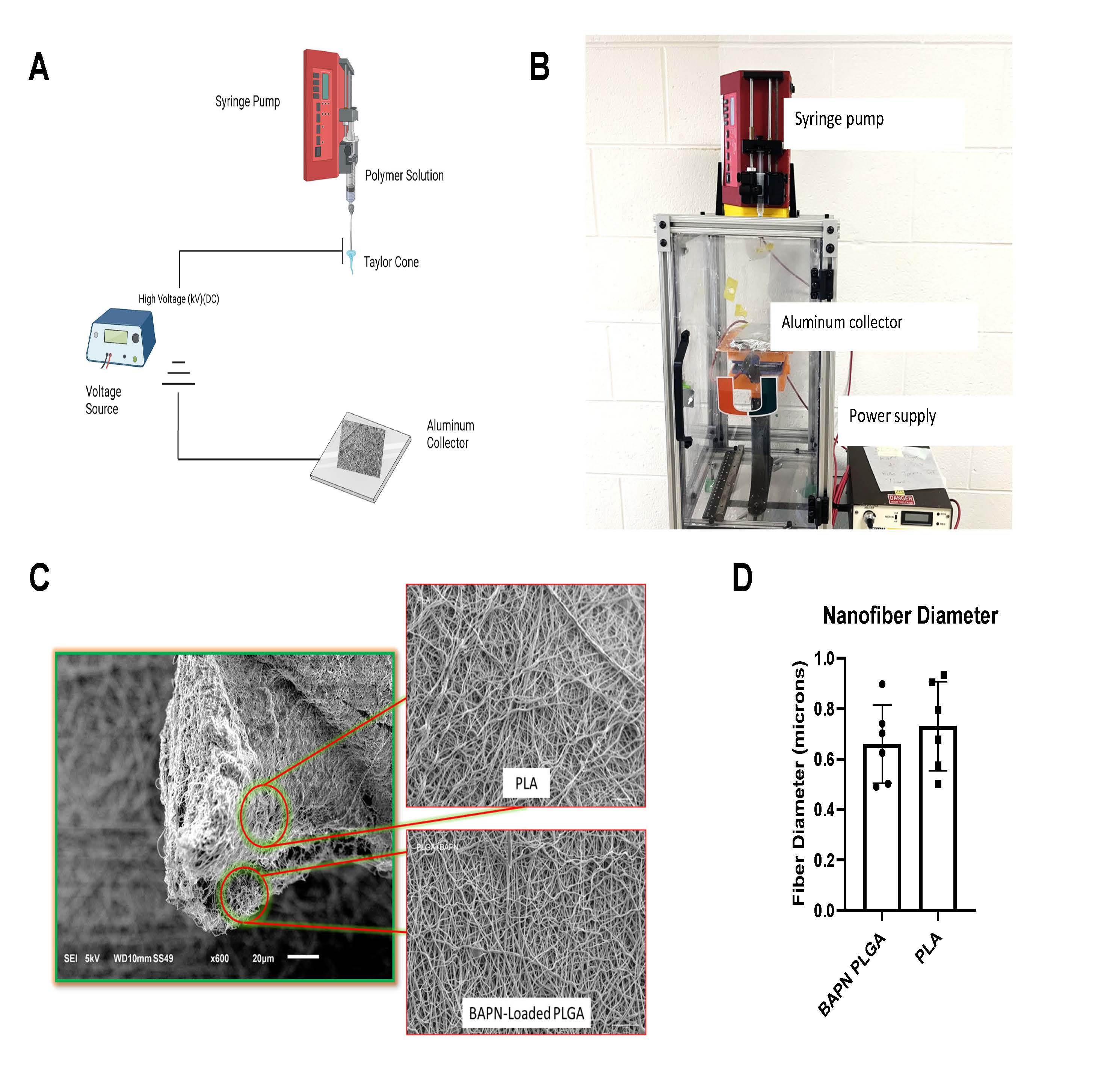

Supplement: Supplementary Figure 2 — Electrospinning of nanofiber scaffolds. (A) Diagram of electrospinning setup. Made with Biorender.com. (B) Picture of our custom electrospinning apparatus. (C) Scanning electron micrograph of bilayer PLA-PLGA nanofiber scaffolds. (D) Average fiber diameter of the PLA and PLGA layers. [file Image_2.JPEG]

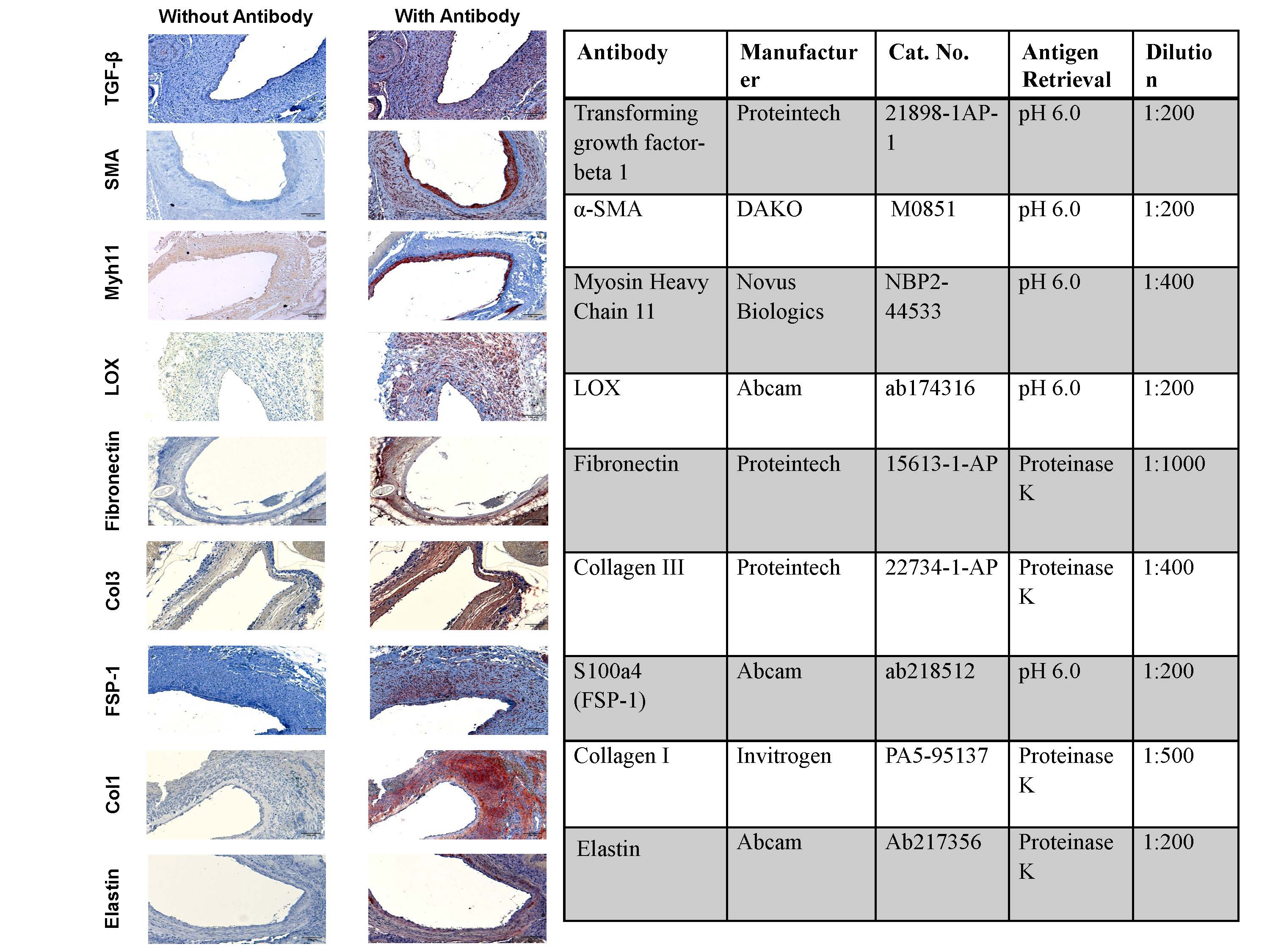

Supplement: Supplementary Figure 3 — Antibodies and dilutions. [file Image_3.JPEG]

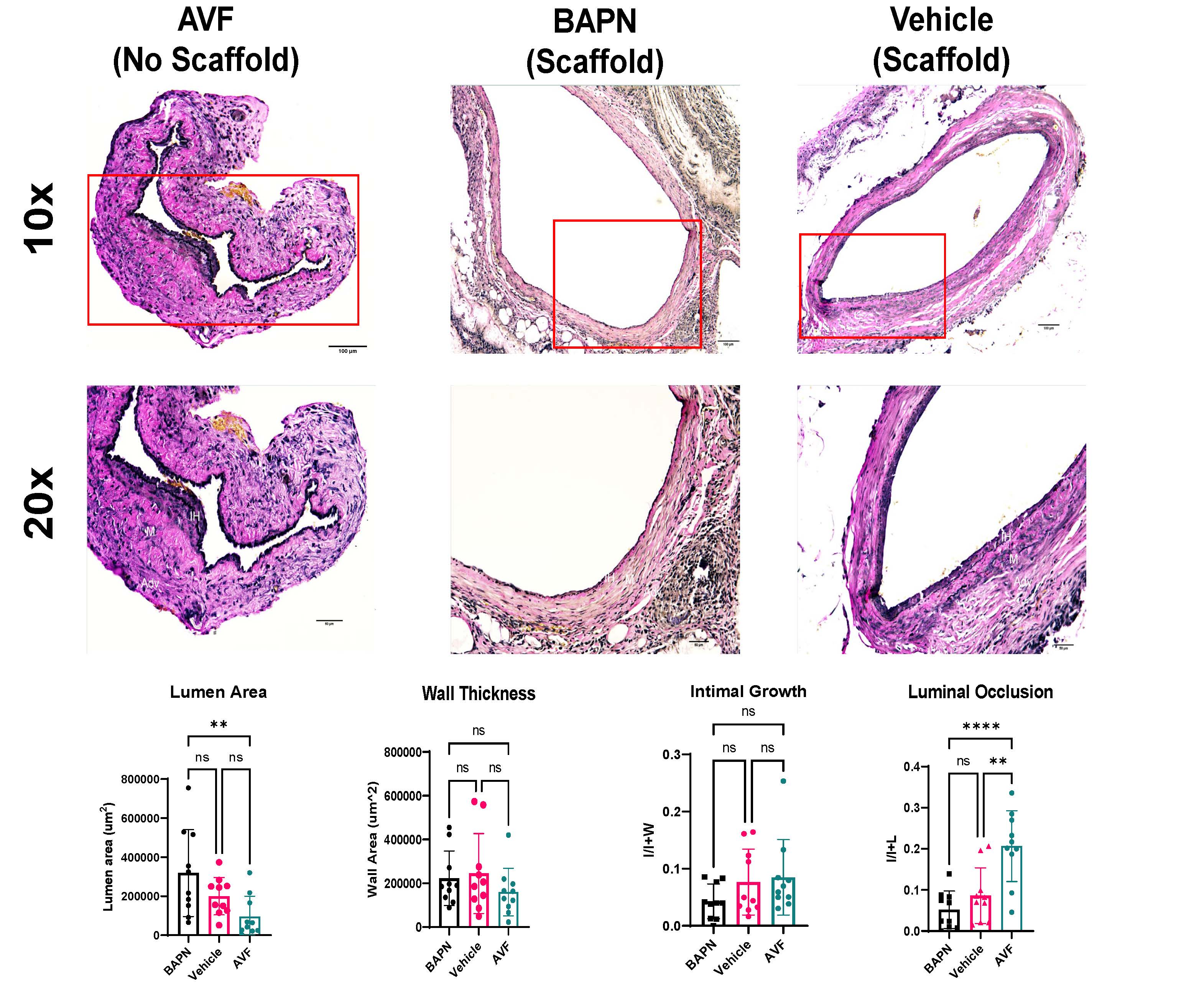

Supplement: Supplementary Figure 4 — Histomorphometry with Sham AVF controls. [file Image_4.JPEG]
